# Serotype Distribution of Clinical *Streptococcus pneumoniae* Isolates before the Introduction of the 13-Valent Pneumococcal Conjugate Vaccine in Cambodia

**DOI:** 10.4269/ajtmh.17-0692

**Published:** 2018-01-08

**Authors:** Malin Inghammar, Youlet By, Christina Farris, Thong Phe, Laurence Borand, Alexandra Kerleguer, Sophie Goyet, Vonthanak Saphonn, Chanleakhena Phoeung, Sirenda Vong, Blandine Rammaert, Charles Mayaud, Bertrand Guillard, Chadwick Yasuda, Matthew R. Kasper, Gavin Ford, Steven W. Newell, Ung Sam An, Buth Sokhal, Sok Touch, Paul Turner, Jan Jacobs, Mélina Messaoudi, Florence Komurian-Pradel, Arnaud Tarantola

**Affiliations:** 1 Institut Pasteur du Cambodge, Phnom Penh, Cambodia;; 2 Section for Infection Medicine, Department of Clinical Sciences Lund, Lund University, Skane University Hospital, Lund, Sweden;; 3 Fondation Mérieux, Phnom Penh, Cambodia;; 4 University of Health Science, Phnom Pen, Cambodia;; 5 Naval Medical Research Unit No. 2, Phnom Penh, Cambodia;; 6 Sihanouk Hospital Center of Hope, Phnom Penh, Cambodia;; 7 CHU de Poitiers, Service de Maladies Infectieuses et Tropicales, INSERM U1070, Université de Poitiers, Poitiers, France;; 8 Cambodian National Laboratory of Public Health, Phnom Penh, Cambodia;; 9 Cambodian Communicable Disease Control Department, Phnom Penh, Cambodia;; 10 Cambodia Oxford Medical Research Unit, Siem Reap, Cambodia;; 11 Nuffield Department of Medicine, Centre for Tropical Medicine and Global Health, University of Oxford, Oxford, United Kingdom;; 12 Department of Clinical Sciences, Institute of Tropical Medicine, Antwerp, Belgium;; 13 Department of Microbiology and Immunology, KU Leuven, Leuven, Belgium;; 14 Emerging Pathogens Laboratory, Fondation Mérieux, Centre International de Recherche en Infectiologie, INSERM U1111, Lyon, France

## Abstract

Childhood vaccination with the 13-valent pneumococcal conjugate vaccine (PCV13) was introduced in Cambodia in January 2015. Baseline data regarding circulating serotypes are scarce. All microbiology laboratories in Cambodia were contacted for identification of stored isolates of *Streptococcus pneumoniae* from clinical specimens taken before the introduction of PCV13. Available isolates were serotyped using a multiplex polymerase chain reaction method. Among 166 identified isolates available for serotyping from patients with pneumococcal disease, 4% were isolated from upper respiratory samples and 80% were from lower respiratory samples, and 16% were invasive isolates. PCV13 serotypes accounted for 60% (95% confidence interval [CI] 52–67) of all isolates; 56% (95% CI 48–64) of noninvasive and 77% (95% CI 57–89) of invasive isolates. Antibiotic resistance was more common among PCV13 serotypes. This study of clinical *S. pneumoniae* isolates supports the potential for high reduction in pneumococcal disease burden and may serve as baseline data for future monitoring of *S. pneumoniae* serotypes circulation after implementation of PCV13 childhood vaccination in Cambodia.

## INTRODUCTION

*Streptococcus pneumoniae* (pneumococci) cause a wide spectrum of infections, ranging from invasive disease with a high case-fatality rate to asymptomatic colonization. Despite available antibiotics, it is estimated that around 800,000 children die every year due to pneumococcal disease, especially in developing countries where timely access to adequate health care is limited.^1^

Pneumococci can be divided into more than 90 different serotypes, based on differences in their capsular polysaccharides, with varying ability to cause severe disease.^2,3^ The distribution of serotypes varies with age and geographical area, whereas the potential coverage rates of vaccines may differ by target population.^4,5^ In high-income countries, the incidence of invasive pneumococcal disease by included vaccine types has declined significantly with the introduction of the pneumococcal conjugated vaccine (PCV).^6,7^ Data from low-income countries are less robust.^8^

In Cambodia, lower respiratory infection is estimated to be the second leading cause of morbidity and mortality.^9^ A new vaccination program of newborns including PCV13 (including serotypes 1, 3, 4, 5, 6A, 6B, 7F, 9V, 14, 18C, 19A, 19F, 23F) was launched in 2015.^10^ Data on the serotype distribution in Cambodia before the vaccine introduction are scarce. Two recent studies from a single center in Siem Reap have assessed the serotype distribution in colonizing and invasive strains in children.^11,12^ Data from other parts of Cambodia and data in adults, however, are lacking. The aim of the present study was to document the serotype distribution of *S. pneumoniae* in Cambodia before the introduction of PCV13 in the national childhood immunization program.

## METHODS

All principal microbiological laboratories in Cambodia were contacted for identification of stored isolates of *S. pneumoniae* from specimens taken before January 2015 (i.e., before the introduction of PCV13). Stored strains were identified at 1) Institut Pasteur du Cambodge (IPC), Phnom Penh, as part of the Surveillance and investigation of Epidemics in South-East Asia (SISEA) project, a prospective study of lower respiratory infections in two provincial hospitals (Takeo and Kampong Cham provinces),^13^ as well as from routine cultures performed from 2006 to 2014; 2) Sihanouk Hospital Center of Hope (SHCH), Phnom Penh, systematically collected as part of a microbiological surveillance program “Surveillance of antimicrobial resistance among consecutive blood culture isolates in tropical settings,” 2008 through 2014; 3) Naval Medical Research Unit No. 2 (NAMRU 2), Phnom Penh, as part of a prospective surveillance study “Surveillance and Etiology of Acute Undifferentiated Febrile Illnesses in Cambodia” (Kandal, Kampong Speu, Kratie, Ratanakiri, Stung Treng, and Svay Rieng), 2005–2014. An overview of the origin of isolates and the participating microbiological laboratories are listed in Table 1. The laboratories at the following hospitals were contacted but none of them had any stored pneumococcal isolates from the study time period: National Pediatric Hospital; Kampong Cham; Takeo; Kampot; Battambang; Siem Reap; Calmette; Khmer Soviet; Kossamak; or Kantha Bopha.

**Table 1:** Origin of isolates and participating microbiological laboratories

Name of institution	Details	Specimen	Time period	Number of samples
Institut Pasteur du Cambodge, Phnom Penh	Surveillance and investigation of Epidemics in South-East Asia project. Prospective study of low respiratory infections in two provincial hospitals (Takeo and Kampong Cham) Ethical approval No. 048-NECHR.^13^	Blood, pleural fluid, sputum	2007–2009	75
	Routine cultures	Blood, bronchoalveolar lavage, sputum	2006–2014	58
Sihanouk Hospital Center of Hope, Phnom Penh	Microbiological Surveillance Study “Surveillance of antimicrobial resistance among consecutive blood culture isolates in tropical settings.” Ethical approvals No: Ethical approval Nos. 009-NECHR and 0313-NECHR.	Blood, cerebrospinal fluid	2008–2014	15
Naval Medical Research Unit No. 2, Phnom Penh	Surveillance and Etiology of Acute Undifferentiated Febrile Illnesses in Cambodia (Kandal, Kampong Speu, Kratie, Ratanakiri, Stung Treng and Svay Rieng). Ethical approval No: NAMRU2.2005.0004.	Sputum, blood, cerebrospinal fluid	2005–2014	18

NECHR = National Ethics Committee in Cambodia.

Information was collected for each contributed sample on the following: date of culture; type of specimen; patient’s date of birth; and on clinical diagnosis and antibiotic susceptibility, if available. All individual information was anonymized before being analyzed. The study was approved by the National Ethics Committee in Cambodia (No. 460-NECHR).

### Serotyping method.

The isolates from the respective participating sites had been processed using standard microbiological procedures at the contributing sites and stored at −80°C. Antimicrobial susceptibility had been determined by disk diffusion at the participating laboratories according to the successive versions of Clinical and Laboratory Standards Institute (CLSI) guideline “Performance Standards for Antimicrobial Susceptibility Testing - Supplement,” CLSI M100-S21 (SHCH, NAMRU 2) and the Antibiogram Committee of the French Society for Microbiology, CA-SFM/EUCAST (IPC).^14^ Results for penicillin, ceftriaxone, cotrimoxazole, and erythromycin were reported as susceptible (S), intermediate (I), or resistant (R). The isolates were typed in the Rodolphe Mérieux Laboratory of the University of Health Sciences, using a multiplex real-time polymerase chain reaction (PCR) method as described previously.^15^ Briefly, DNA was extracted directly from 100 μL stored isolates using an easyMAG automate (bioMérieux, Lyon, France) according to the manufacturer’s recommendation and then typed using a panel of multiplex PCR, enabling the detection of the 40 most prevalent *S. pneumoniae* serotypes worldwide and including an internal positive control targeting the lytA gene, a gene conserved among pneumococci. A subset of the isolates from the SHCH were serotyped using latex agglutination method with Quellung confirmation of ambiguous results at Angkor Hospital for Children/Cambodia Oxford Medical Research Unit microbiology laboratory.^16,17^

### Data analyses.

The pneumococcal serotypes were grouped into PCV13 vaccine types (1, 3, 4, 5, 6A, 6B, 7F, 9V, 14, 18C, 19A, 19F, 23F) and nonvaccine types (all others, including non-typeable). Groups were compared using a Wilcoxon Rank Sum test, χ^2^, or Fisher’s exact test, as appropriate. The distribution of serotype groups in invasive isolates (blood, cerebrospinal fluid [CSF], and pleural fluid) and noninvasive isolates (all other isolates) were compared. In case of multiple samples per patient and index date, only the first isolate was included. A sensitivity analysis was performed, including only isolates originating from prospective studies or prospective systematic sample collection (IPC-SISEA, NAMRU 2 and SHCH). Analyses were made using Stata SE, version 13.1 (StataCorp, College Station, TX).

## RESULTS

In total, 249 isolates were identified at the participating institutions: 215 from IPC, 16 from SHCH, and 18 from NAMRU 2. Of these, we were unable to determine the serotype of 79 (32%) isolates because of sample contamination or degradation, four isolates were excluded as they stem from the same patient and index date, leaving 166 (67%) isolates in the analysis. Of these, 133 (80%) came from IPC, 15 (9%) from SHCH, and 18 (11%) from NAMRU 2. The basic characteristics of the isolates are shown in Table 2. Information on sex was available for 106/166 isolates.

**Table 2:** Basic characteristics of the patients

Total number	166 (100)
Age in years, median (range)	39 (1–84)
Age groups, *N* (%)	
0–15 years	36 (22)
16–65 years	101 (61)
> 65 years	29 (17)
Male sex,* *N* (%)	55 (52)
Time of sampling, *N* (%)	
Rainy season (May–October)	79 (48)
Dry season (November–April)	87 (52)

* Information on sex was available for 106/166 isolates.

Twenty-six (16%) were invasive isolates (blood, CSF, and joint fluid); six (4%) were isolated in upper respiratory samples (ear, eye, and nasopharyngeal swab) and 133 (80%) were from lower respiratory samples (bronchoalveolar lavage fluid or sputum). Table 3 shows the serotype distribution per sample type.

**Table 3:** Serotype distribution per specimen

	Total number of samples	Samples included in PCV13* number (%)	Serotypes (*n*)†
Blood	21	18 (86)	**1** (4), **14** (3), **19A** (3), **19F** (3), 23A (1), **23F** (2), 34 (1), 38 (1), **6AB** (1), **7F** (1), **9V** (1)
Cerebrospinal fluid	4	1 (25)	**18C** (1), 24F (2), 34 (1)
Joint fluid	1	1 (100)	**23F** (1)
Upper respiratory samples‡	6	4 (67)	11A (1), **14** (1), **19A** (2), **19F** (1), **23F** (1), 35B (1)
Lower respiratory samples§	133	74 (56)	10A (1), 11A (2), 12F (1), 13 (4), **14** (4), 15A (2), 15BC (4), 17F (1), 18 (1), **19A** (5), **19F** (25), 20 (1), 21 (1), 22F (2), **23F** (22), **3** (1), 34 (20), 35A (4), 35B (2), 35F (4), 38 (1), **6AB** (16), 6C (2), **9V** (1), non-typeable (6)
Total	166	99 (60)	**1** (4), 10A (1), 11A (3), 12F (1), 13 (4), **14** (8), 15A (2), 15BC (4), 17F (1), 18 (1), **18C** (1), **19A** (10), **19F** (29), 20 (1), 21 (1), 22F (2), 23A (1), **23F** (26), 24F (2), **3** (1), 34 (22), 35A (4), 35B (3), 35F (4), 38 (2), **6AB** (17), 6C (2), 7F (1), **9V** (6), non-typeable (6)

* Serotypes included in PCV13—13-valent pneumococcal conjugate vaccine.

† Serotypes included in PCV13 are marked in bold.

‡ Upper respiratory samples: ear, eye, and nasopharyngeal swab.

§ Lower respiratory samples: sputum or bronchoalveolar fluid.

**Figure 1.**
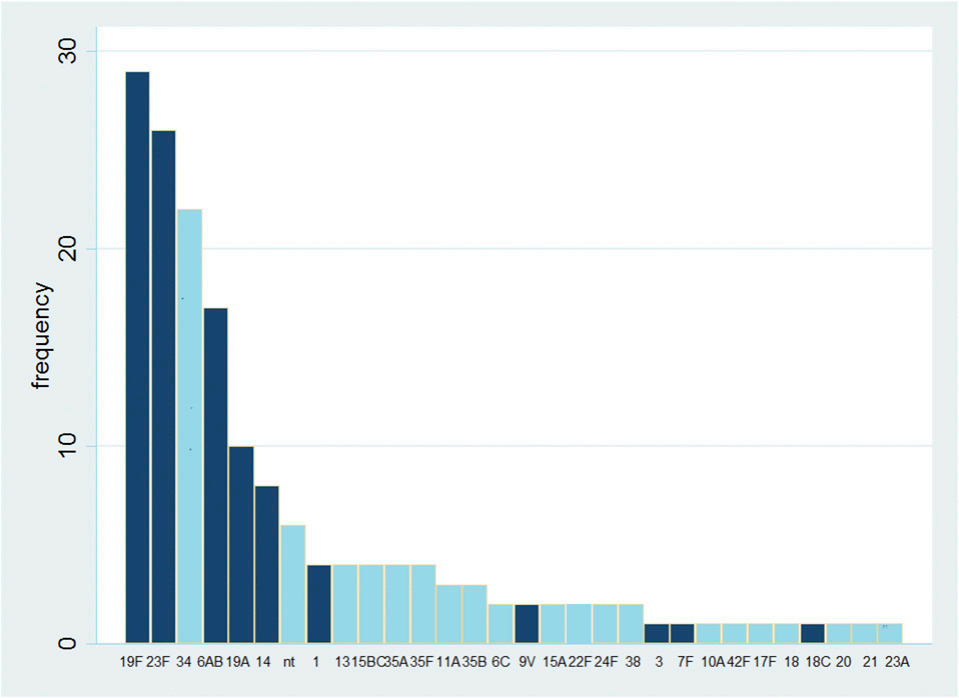
Serotype distribution of 166 isolates cultured from samples collected from 2006 to 2014 in Cambodia. Serotypes included in 13-valent pneumococcal conjugate vaccine are shaded in dark. Non-typeable strains are denoted “nt.” This figure appears in color at www.ajtmh.org.

The serotype distribution is shown in Figure 1. The three most common serotypes were 19F, 29/166 (17%), 23F, 26/166 (16%), and 34, 22/166 (13%).

Overall, PCV13 serotypes accounted for 60% (95% confidence interval [CI] 52–67) of the isolates (Table 3). Among noninvasive isolates, the proportion of PCV13 serotypes was 56% (95% CI 48–64) and among invasive isolates the proportion was 77% (95% CI 57–89) (*P* = 0.05). The proportion of PCV13 serotypes was similar between age groups: 25 (70%) among 36 isolates from patients ≤ 15 years of age; 59 (58%) among 101 isolates from patients 16–64 years old; and 15 (52%) among 29 isolates from patients aged 65 years or greater (*P* = 0.32).

In the sensitivity analyses among 108 isolates systematically collected as part of surveillance studies (NAMRU 2, SHCH, IPC-SISEA), the proportion of PCV13 serotypes was 50% (95% CI 41–60) overall; 42% (95% CI 32–53) and 76% (95% CI 55–89) among noninvasive and invasive isolates, respectively.

Data on antibiotic susceptibility were available for 127–165 of the isolates. Of these, 95/165 (58%) were reported susceptible for penicillin G and 70/165 (42%) were reported nonsusceptible (intermediate or resistant); 127/137 (77%) were reported susceptible to amoxicillin, 149/157 (95%) were reported susceptible to ceftriaxone; 15/164 (9%) were reported susceptible to cotrimoxazole; and 81/165 (49%) were reported susceptible to erythromycin (Table 4). Thirty-one percent of the isolates were reported nonsusceptible to three or more of these antibiotics. Multidrug antibiotic resistance was more common among PCV13-covered serotypes than among nonvaccine serotypes, the proportion of PCV13 coverage was 23% among the isolates with no reported resistance to these antibiotics; 59% among isolates with resistance to one or two of the antibiotics; and 71% among isolates with resistance to at least three of the antibiotics (*P* = 0.006).

**Table 4:** Antimicrobial susceptibility data of the isolates according to vaccine coverage of serotypes

	Overall *N* (%)	PCV13 isolates *N* (%)	Non-vaccine isolates *N* (%)	*P* value
Penicillin G*				0.02
Susceptible	95 (58)	50 (51)	45 (68)	
Amoxicillin†				0.31
Susceptible	127 (93)	68 (91)	59 (95)	
Ceftriaxone‡				0.10
Susceptible	149 (94)	87 (93)	62 (98)	
Cotrimoxazole§				0.005
Susceptible	15 (9)	4 (4)	11 (17)	
Erythromycin§				0.006
Susceptible	81 (49)	40 (40)	41 (62)	

PCV13 = 13-valent pneumococcal conjugate vaccine.

* Antibiotic susceptibility testing data were available for 165/166 isolates.

† 157/166 isolates tested.

‡ 164/166 isolates tested.

§ 165/166 isolates tested.

## DISCUSSION

Data from this retrospective study of clinical pneumococcal isolates in Cambodia, collected before the introduction of PCV13 in 2015, suggest that this vaccine potentially covers around 80% of the invasive isolates and around 60% of the noninvasive isolates. The range of serotypes and predicted vaccine coverage is consistent with the two studies previously published. A survey (2013–2014) reported 63% PCV13 coverage in colonizing isolates in pediatric outpatients (*N* = 601) in Cambodia,^12^ and 88% among invasive isolates (*N* = 40). A second study from the same center based on invasive isolates (*N* = 50) 2008–2012 predicted 92% PCV13 coverage.^11^ Furthermore, a review of studies from neighboring countries in Southeast Asia, including both pediatric and adult data, estimated that PCV13 provides 46–72% coverage for the circulating isolates.^18^

Interestingly, serotype 34, which is not included in PCV13, was the third most common serotype. This result contrasts with that of a colonizing survey of children from Siem Reap, Cambodia, where the prevalence of serotype 34 was found low.^12^ Serotype 34 was not found to play a major role in any of the 25 studies reviewed of pneumococcal disease in Southeast Asia. Notably, serotype 34 is believed to be less invasive,^3,19^ and the studies reviewed focused mainly on invasive disease. This could have affected the differences in importance attributed to serotype 34. Because of the limited sample size, it is not possible to infer whether the difference is due to a true increased prevalence compared with neighboring countries or due to sampling variation. Nevertheless, this serotype may become important in the post-PCV introduction era.

The frequency of antibiotic resistance was high in our sample set; overall 60% of the isolates were penicillin-susceptible. PCV13-covered serotypes were significantly less susceptible to penicillin, cotrimoxazole and erythromycin, as compared with the nonvaccine serotypes, whereas there was no significant difference in the frequency of susceptibility to amoxicillin or ceftriaxone. Furthermore, PCV13-covered serotypes were more likely to express resistance to more than one antibiotic class than non-PCV13 serotypes. This finding further supports a potential benefit of PCV13, reducing the incidence of infections caused by penicillin-resistant pneumococci or multidrug-resistant pneumococci.

This study has many limitations. The pre-hospitalization use of antibiotics is widespread in Cambodia, which explains why microbiological yield in cultures is generally low.^12^ Furthermore, we relied on retrospective data on antimicrobial susceptibility testing collected over a long timeframe and at different centers; despite internal and external quality management at the participating laboratories, we cannot exclude errors at the time of assessment. Pre-hospitalization antimicrobial use effective against susceptible nonvaccine serotypes could potentially have biased our results toward a higher proportion of antimicrobial nonsusceptible serotypes. Despite researchers’ efforts to contact all available microbiological laboratories in Cambodia, the study size remained small, with a limited number of invasive isolates. None of the public microbiology laboratories store cultured isolates. We were, however, able to identify isolates from patients with severe pneumococcal infections from five different geographical regions in Cambodia, prospectively and systematically collected as part of well-conducted epidemiological studies on lower and/or severe respiratory infections. We, therefore, are confident that our results may be still generalizable to the general population. Furthermore, results were very similar to the two previously published studies from Cambodia, despite differences in the targeted age group and geographical areas. Importantly, the present study is the first to include adult data.

It has clearly been demonstrated in high-income countries that the incidence of invasive pneumococcal disease due to serotypes included in PCV significantly decreased with the introduction of the vaccine into the childhood vaccination schedule due to a direct effect in vaccinated children as well as an indirect herd effect among older age groups.^6^ However, after the introduction of the vaccine, an increase of nonvaccine serotypes by either selection or replacement has been observed in many countries.^3^ If nonvaccine serotypes carry antibiotic resistance genes, serotype shifting may potentially lead to an increase of the prevalence of antibiotic-resistant pneumococcal clones.^20^ For these reasons, pre-vaccination data are needed to monitor effects of the introduction of PCV. In low-income settings, data are scarce and accurate prediction is often hampered by low-quality epidemiological, biological, or clinical data. Despite the limited study size, our study based on isolates prospectively collected as part of either epidemiological studies or routine care, from patients with severe pneumococcal infections of all ages and from five geographical regions, provides a good baseline pre-vaccination assessment of the epidemiology of pneumococcal strains in Cambodia.

In conclusion, our study supports the potential for high reduction in pneumococcal disease burden with the introduction of PCV13 in childhood vaccination program. Multidrug resistance was higher among strains included in PCV13, which further supports likely vaccine impact of the now implemented vaccine program.
